# Source Localization and Classification of Pulmonary Valve-Originated Electrocardiograms Using Volume Conductor Modeling with Anatomical Models

**DOI:** 10.3390/bios14100513

**Published:** 2024-10-21

**Authors:** Kota Ogawa, Akimasa Hirata

**Affiliations:** Department of Electrical and Mechanical Engineering, Nagoya Institute of Technology, Nagoya 466-8555, Japan

**Keywords:** diagnosis, electrocardiogram, source localization, wearable sensing

## Abstract

Premature ventricular contractions (PVCs) are a common arrhythmia characterized by ectopic excitations within the ventricles. Accurately estimating the ablation site using an electrocardiogram (ECG) is crucial for the initial classification of PVC origins, typically focusing on the right and left ventricular outflow tracts. However, finer classification, specifically identifying the left cusp (LC), anterior cusp (AC), and right cusp (RC), is essential for detailed preoperative planning. This study aims to improve the accuracy of cardiac waveform source estimation and classification in 27 patients with PVCs originating from the pulmonary valve. We utilized an anatomical human model and electromagnetic simulations to estimate wave source positions from 12-lead ECG data. Time-series source points were identified for each measured ECG waveform, focusing on the moment when the distance between the estimated wave source and the pulmonary valve was minimal. Computational analysis revealed that the distance between the estimated wave source and the pulmonary valve was reduced to less than 1 cm, with LC localization achieving errors under 5 mm. Additionally, 74.1% of the subjects were accurately classified into the correct origin (LC, AC, or RC), with each origin demonstrating the highest percentage of subjects corresponding to the targeted excitation origin. Our findings underscore the novel potential of this source localization method as a valuable complement to traditional waveform classification, offering enhanced diagnostic precision and improved preoperative planning for PVC ablation procedures.

## 1. Introduction

Heart diseases remain a leading cause of mortality worldwide, necessitating continual advancements in diagnostic and therapeutic techniques [1]. Arrhythmias often evade detection through imaging diagnostics, such as echocardiography or MRI, due to the absence of structural abnormalities, making electrocardiogram (ECG) diagnosis invaluable [2]. The 12-lead ECG, a cornerstone in clinical practice, monitors electrical potentials through six chest and three limb electrodes, creating 12 distinct vector patterns [1].

Premature ventricular contractions (PVCs) are a common arrhythmia characterized by ectopic excitations within the ventricles. Standard treatment involves catheter ablation, where the ectopic focus is identified and ablated to prevent recurrence [3,4,5]. While ECG is crucial for the initial classification of the PVC origin, the precise localization of the ablation site still relies heavily on intraoperative exploration using catheter electrodes [4]. This approach is time-consuming, burdensome for patients, and highly dependent on the operator’s skill [6]. As a result, developing accurate, ECG-based preoperative techniques is urgently required for estimating cardiac sources.

PVCs can originate from outflow tracts, most arising from the right or left ventricular outflow tracts (RVOT or LVOT) [7,8,9,10,11]. Further simple classifications, such as normal or abnormal, are also considered [12,13]. Previous studies have primarily focused on classifying the origin as RVOT or LVOT using machine learning or identifying ectopic excitations targeting these outflow tracts [14,15]. The results can provide high accuracy for classifying these two targets. However, for clinical purposes, further classification is desirable for operational planning, mainly left cusp (LC), anterior cusp (AC), and right cusp (RC) origins [16,17,18], but such studies are limited.

Additionally, computational studies have utilized anatomically based human body models for source estimation in the specific context of cardiac diagnosis [19,20,21,22]. These studies have made significant advances in using personalized models for accurate simulation and localization, particularly in atrial and ventricular arrhythmias. However, ECG-based source estimation for PVCs originating at the pulmonary valve remains unexplored. Precise localization and classification in these cases are clinically valuable for reducing diagnostic time and improving operational planning. ECG-based source estimation has not yet been applied to subjects with PVCs originating at the pulmonary valve. Accurate localization and classification in these cases would be clinically valuable, particularly in reducing the time needed for diagnosis and improving operational planning.

This study addresses a critical gap in the preoperative estimation of PVC origins, particularly those arising from the pulmonary valve, which existing methods have largely overlooked. We aimed to estimate the sources of cardiac waveforms in patients with PVCs originating from the pulmonary valve. To achieve this, we utilized detailed human models to perform source estimation of ectopic excitations using 12-lead ECG waveforms from 27 patients. We assessed the accuracy of our source estimation method for cardiac waveforms originating at the pulmonary valve. We further explored the classification of these ablation origins by calculating the Euclidean distance between the estimated wave source positions and the actual ablation sites. ECG monitoring with wearable sensors is becoming increasingly prevalent [23,24,25], and its potential for enhanced diagnostic applications is anticipated. The proposed approach, utilizing a single standard human body model, could prove valuable in estimating the origin of ECG signals for broader diagnostic purposes.

## 2. Method

In this study, we use previously reported experimental data to replicate source localization and classification using a numerical approach. Section 2.1 outlines the experimental scenarios in detail. The remainder of this section focuses on the computational replication based on the experimental data presented in Section 2.1. Specifically, for a standardized human body model in Section 2.2, electrical phenomena, such as EEG, are replicated within the computational domain for the electromagnetics method described in Section 2.3. The source location in the heart is then estimated by solving an inverse problem in Section 2.4. For the scenarios, settings, and procedures in Section 2.5 and Section 2.6, the source origin was estimated in the time domain.

### 2.1. Data Source and Its Acquisition Scenario

We have stated that the original dataset contains 334 subjects who successfully underwent catheter ablation [26], which validated the accurate origins of idiopathic ventricular arrhythmia. From this dataset, we selected 27 patients whose anatomical sources were identified as LC, AC, and RC.

The ablation procedure was as follows: Antiarrhythmic medications were discontinued at least 5 half-lives before the procedure. The ablation was performed under the guidance of fluoroscopy and a three-dimensional electroanatomic mapping system. Activation mapping was conducted during VT or PVC episodes, and pace mapping was performed when VT or PVCs were infrequent, using a pacing cycle length of 500 ms and the minimum stimulus amplitude required for consistent capture.

Maximum radiofrequency energy was delivered with a power of up to 50 W and an electrode–tissue interface temperature of 55 °C. If VT or PVCs diminished or disappeared after 30 s of ablation, the energy was applied continuously for 60 to 180 s. Ablation success was defined as the absence of spontaneous or induced outflow tract ventricular arrhythmias 30 min after the last energy delivery.

**Figure 1:**
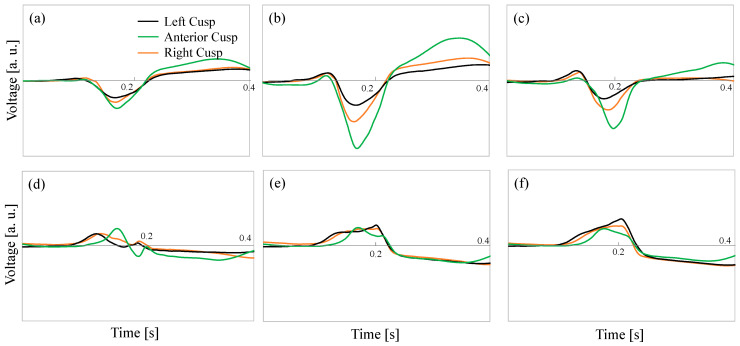
Examples of ECG waveforms (voltage) for the origins from left cusp (LC), anterior cusp (AC), and right cusp (RC). (**a**) V1, (**b**) V2, (**c**) V3, (**d**) V4, (**e**) V5, and (**f**) V6.

For post-procedural monitoring, success was confirmed through continuous cardiac telemetry over the next 24 h of inpatient care. Typical ECG waveforms for the origins from LC, AC, and RC are shown in Figure 1.

### 2.2. Human Body Model

An anatomical numerical human body model (XCAT) developed by Duke University Medical Center was used [27]. XCAT is a voxel model with a resolution of 1 mm created based on CT images, comprising 18 types of biological tissues, including skin, muscles, fat, bones, and heart. The heart tissue in XCAT is simplified, lacking a clear separation between atria and ventricles, and is represented as a single tissue. Different conductivities for 17 biological tissue types, excluding the skin, were assigned according to the 4-Cole–Cole dispersion model [28], with a conductivity of 0.1 S/m for the skin [29]. Minor tuning has been carried out for this purpose, as per our previous study [30]. 

### 2.3. Volume Conductor Model

Displacement currents are negligible given the low frequency of ECG waveforms (~1 Hz) [1,24]. We used the scalar-potential finite-difference (SPFD) method [31,32] to efficiently compute the electric field in a voxel-based body model, addressing the computational intensity associated with the finite element method analysis [21].

The scalar potential *ϕ* in the volume conductor is governed by the Poisson equation:

(1) $$\left\{\begin{array}{c} \nabla \cdot \left(\sigma \nabla \phi \right)=-\nabla \cdot J\;\mathrm{in}\;\Omega \;\\ \left(\sigma \nabla \phi \right)\cdot n_{B}=0\;\mathrm{on}\;B_{S} \end{array}\right.,$$

where *σ*, ***J***, ***n****_B_*, *Ω*, and *B_S_* are the tissue electrical conductivity, current density, a unit vector outwardly normal to the body surface, volume conductor, and body surface, respectively. This equation was discretized using a quasi-static approximation [25]:

(2) $$\left(\sum_{n=1}^{6}S_{n}\right)\phi_{0}-\sum_{n=1}^{6}S_{n}\phi_{n}=-j\omega q,$$

where *n* represents the node index, *ϕ_n_* is the electric potential at the *n*-th node, ω is the angular frequency, *q* is the electric charge at the central node, and *S*_n_ is the conductance between the *n*-th node and the central node, calculated based on the conductivity of the surrounding tissue voxels.

Boundary conditions were enforced by setting air voxels adjacent to the body surface. The resulting equations were solved using Kirchhoff’s current law and an iterative process, enhanced by the successive over-relaxation method [27] and a multigrid method [28]. Six multigrid levels were used to expedite the solution, with computations continuing until the relative residual fell below 10^−6^.

### 2.4. Lead Field Matrix

The position of the cardiac electrical sources was estimated using a lead field matrix (LFM) [7], known for solving inverse problems with high accuracy. The coefficient matrix representing the relationship between the current density of equivalent current sources in the heart tissue and the observed potential is expressed in Equation (3):

(3) L⋅j=Φ,

where L is an LFM of size M × 3N, j is a current density vector of size 3N × 1, and Φ is an observed potential vector of size M × 1. Electric dipoles simulating the heart’s electrical activity were placed in the basal direction of the heart tissue for the forward problem to generate LFM. The observed potential was normalized by the dipole moment direction of the positive charge position, and the column vectors of LFM were obtained [14].

### 2.5. Scenarios

Nine electrodes were placed based on the standard 12-lead ECG [1]. LFM was constructed by performing forward problem analysis for the basal three directions next to the estimation target in the heart tissue. An orthogonal matching pursuit (OMP) method estimated the cardiac wave source positions [15]. This algorithm sequentially selects column vectors from LFM to reconstruct the elements of the estimated vector, starting from the one with the maximum correlation coefficient with the residual. The estimation algorithm is presented in Algorithm 1. The iteration was performed five times, and a weighted average using the updated residual was used to calculate one estimated current density vector for any input potential.


**Algorithm 1** Pseudocode of the proposed algorithm using orthogonal matching pursuitInitialize current density vector: $\hat{j}=0$
Initialize support vector: $\hat{L}$
**= 0**
for i=1:3N


$corrcoef(i)=\frac{\phi \cdot L_{i}}{\left\|\phi \right\|\left\|L_{i}\right\|}$

end forEstimated source location: $\hat{i}=\underset{i}{\mathrm{argmax}}corrcoef(i)$
Support vector: $\hat{L}=[\hat{L},L_{\hat{i}}]$
Reconstruction of current density: $\hat{j}={\hat{L}}^{+}\cdot \phi$
Update the residual: $\phi =\phi -\hat{L}\cdot \hat{j}$



**Figure 2:**
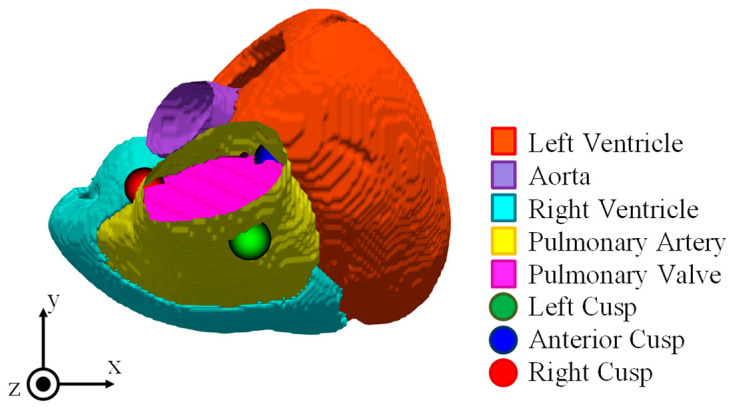
Ventricular structure and location of pulmonary artery valve.

The input potentials were the standard 12-lead ECG waveforms of patients with PVCs [16]. PVCs are classified in detail based on the origin of abnormal excitations, using the position relationship of blood vessels and valves as references. Twenty-seven patients with PVCs originating from the pulmonary valve of the RVOT were studied, including fifteen with LC, six with AC, and six with RC origin (Figure 2). Waveform processing included noise removal using the wavelet transform applied to the dataset, and DC offset removal by dividing with the baseline. The sampling frequency was 2 kHz, and the time-series data of the input potentials were taken from the rise to the peak of the ECG waveform.

### 2.6. Analysis Procedure

**Figure 3:**
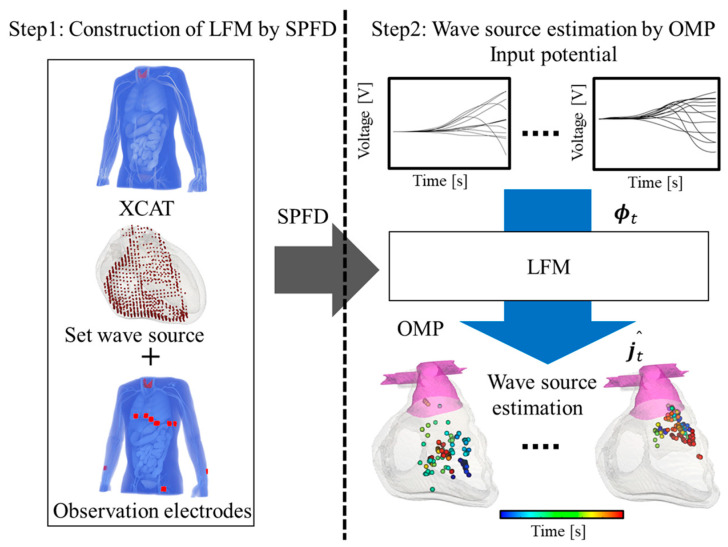
Flowchart of analysis and estimation. Electrodes (observation points) are set on the XCAT human body model. Then, the wave source is assumed to be distributed in the heart volume for the inverse problem. Then, the LFM is constructed from scalar potential and finite difference (SPFD) method. From the measured data, the wave source is estimated in the time domain.

A flowchart of the analysis and estimation is presented in Figure 3. First, electric dipoles were set as wave sources on the numerical human body model, and electromagnetic field analysis was performed using the SPFD method. Then, LFM was constructed using the observed potential and the distribution of internal current density obtained from the forward problem analysis. Furthermore, for a given time, the OMP method was applied to LFM to calculate the current density vector j, and the estimated wave source for each time was determined by repeating this for each time step. Our previous studies verified the analysis procedure for healthy subjects’ data [30,33,34]. The source localization following this procedure estimates the location of the source at each time step.

The minimum Euclidean distance between the source location at each time step and the corresponding target regions (AC, RC, and LC) is calculated. The target region with the minimum distance is classified as the origin of the source. What distinguishes our approach from other studies is that the analysis is conducted in the time domain during the post-processing stage. The minimum distance from each potential origin (AC, RC, and LC) is identified over time, and the region with the overall minimum distance is classified as the origin, i.e., AC, RC, or LC.

## 3. Results

**Figure 4:**
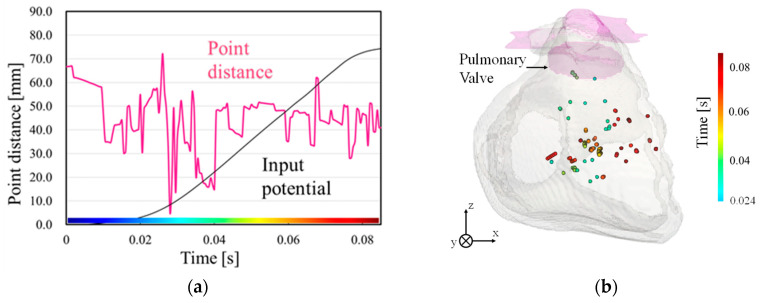
Example of wave source estimation results. (**a**) Minimum Euclidean distance between estimated point and pulmonary valve, and (**b**) estimated source location in the time domain of wave source and pulmonary valve from 0.024 s to 0.082 s. The distance was calculated for different target regions. The region with the minimum distance was classified as the source origin.

The estimated wave source for one subject is presented in Figure 4 as an example. The time point when the Euclidean distance between the estimated wave source positions and the pulmonary valve is minimal corresponds to a temporally localized period during the rise of the input waveform (Figure 4a). Before this estimated target time, the small amplitude observed during the early excitation phase may cause significant estimation errors. Therefore, the analysis focuses on excitation propagation from this critical time point. Figure 4b demonstrates that excitation begins at this time and spreads to the right ventricle, indicating that the estimation aligns with the excitation mechanism observed in PVCs. At 0.025 s, a few plots appear around pulmonary valve, corresponding to the origin in RC. The minimum point distance between the plot and RC was 5.23 mm.

Table 1 summarizes the average minimum Euclidean distance between the estimated origin of excitation and the pulmonary valve across the 27 subjects. The table confirms that for LC and RC origins, there is an estimated point with a minimum distance of less than 10 mm from the pulmonary valve, while the distance is more considerable for AC.

The minimum Euclidean distance from the origins LC, AC, and RC to the pulmonary valve, the site of abnormal excitation, was calculated and compared for origin classification. Four out of six subjects (66.7%) with RC as the origin had the minimum Euclidean distance associated with RC. Twenty out of twenty-one subjects (95.2%) with LC or AC as the origin had the minimum Euclidean distance associated with LC. This is likely due to the similarity in excitation propagation between LC and AC [17] and the smaller amount of myocardial tissue near AC. However, differences were observed in the excitation sequence of myocardial tissue near each origin (LC, AC). The classification was performed by comparing the timing at which each origin exhibited the minimum Euclidean distance to account for the abovementioned differences. The excitation sequence was applicable in 11 out of 14 subjects with LC as the origin (78.6%) and in 5 out of 6 subjects with AC as the origin (83.3%), successfully classifying 16 out of 20 subjects (80.0%).

Table 2 presents the classification results for subjects with the pulmonary valve as the origin. The table indicates that among the 27 subjects with the pulmonary valve as the origin, 20 (74.1%) were correctly classified as the target origin. Additionally, the highest percentage of targeted excitation origin for each subject was confirmed, highlighting the effectiveness of the origin classification for cardiac waveforms originating from the pulmonary valve.

## 4. Discussion and Concluding Remarks

This study conducted wave source estimation for the cardiac waveforms of 27 patients with the pulmonary valve as the origin. The study aimed to demonstrate that source localization could provide valuable insights in addition to waveform classification. Despite the increasing use of machine learning for waveform classification, studies focusing on the classification of LC, AC, and RC origins have been limited.

Through electromagnetic computer simulations, we confirmed moments when the distance between the estimated wave source position and the pulmonary valve was small. The error was generally less than 1 cm using the minimum distance in the time domain, with LC localization achieving an error of less than 5 mm. Comparing our results with previous studies is challenging, as no study has explored these specific classifications or the same target areas. Recent studies using anatomically realistic human body models have reported mean localization errors was 12.6 ± 11.35 mm [33] and 10.13 ± 5.13 mm [35] for different targets. The estimation error in this study (Table 1) is comparable to or better than those in previous studies. Variations in waveform considerations across different studies may also affect localization accuracy. For example, the necessity of offset correction for signals has been suggested as an important factor [36], and this study also considered it.

Subsequently, we explored the classification of cardiac waveforms originating from the pulmonary valve. In 74.1% of the subjects, they were classified into the target origin, and for each origin, the percentage of subjects with the targeted excitation origin was the highest; the highest rate of subjects corresponded to the targeted excitation origin. These findings suggest the effectiveness of the wave source estimation method and the origin classification for cardiac waveforms originating from the pulmonary valve. The origin estimation’s accuracy would be improved if combined with machine learning for waveform classification.

Future research should address individual differences by incorporating body size into the model and enhancing the accuracy of the estimation algorithm by applying correction methods for time-series data. Additionally, for a standardized body model, achieving efficient localization with low computational cost would be essential for wearable diagnostic applications.

## Figures and Tables

**Table 1:** Minimum Euclidean distance from pulmonary valve. SD represents standard deviation.

Subjects	Minimum Point Distance (Mean ± SD [mm])
LC (*n* = 15)	4.59 ± 8.54
AC (*n* = 6)	10.42 ± 12.45
RC (*n* = 6)	9.06 ± 6.00

**Table 2:** Classification results in subjects of pulmonary artery origin.

Subject	Origin in the Pulmonary Valve		
	LC	AC	RC
LC (*n* = 15)	73.3% (*n* = 11)	20.0% (*n* = 3)	6.7% (*n* = 1)
AC (*n* = 6)	16.7% (*n* = 1)	83.3% (*n* = 5)	0%
RC (*n* = 6)	33.3% (*n* = 2)	0%	66.7% (*n* = 4)

## Data Availability

Raw data in this study are available upon reasonable request.
